# Severity of blood flow reduction associated with detectable T2 enhancement in the area at risk of a reperfused canine model of acute myocardial infarction

**DOI:** 10.1186/1532-429X-16-S1-M10

**Published:** 2014-01-16

**Authors:** Sophia Hammer-Hansen, Li-Yueh Hsu, Steve W Leung, Joni Taylor, Martin Ugander, Andrew E Arai

**Affiliations:** 1 National Heart, Lung, and Blood Institute, Bethesda, Maryland, USA; 2 University of Kentucky, Lexington, Kentucky, USA; 3 Karolinska Institute and Karolinska University Hospital, Stockholm, Sweden

## Background

T2-weighted enhancement depicts the myocardial area at risk (AAR) associated with coronary occlusion. However, the severity of blood flow abnormality required to cause a detectable elevation in myocardial T2 is unknown. The aim of this study was to use microspheres to determine the severity of reduction in myocardial blood flow that results in visually detectable T2 weighted enhancement.

## Methods

Surface coil intensity corrected T2 prepared SSFP at 1.5T was performed after 2 hours of coronary occlusion followed by 4 hours of reperfusion in a canine model. Myocardial blood flow during ischemia was determined by administering microspheres. A mid-ventricular short axis slice of the heart was divided into 16 transmural sectors for microsphere analysis. MR images were matched to the pathological slices at the sector level using papillary muscles and RV insertion as landmarks. ROIs delineated T2-enhanced and remote regions of the MR images and measurements were compared with microsphere blood flows. Results were reported as median (interquartile range [IQ]) and compared using the signed Wilcoxon rank test. Each T2-prepared sector was classified as bright (AAR) or normal (remote) by 2 independent readers and compared with absolute microsphere blood flow and flow relative to remote myocardium. ROC analyses were performed to find the thresholds of absolute and relative blood flow reduction needed to detect T2 enhancement in the sector based analysis.

## Results

**Figure 1:**
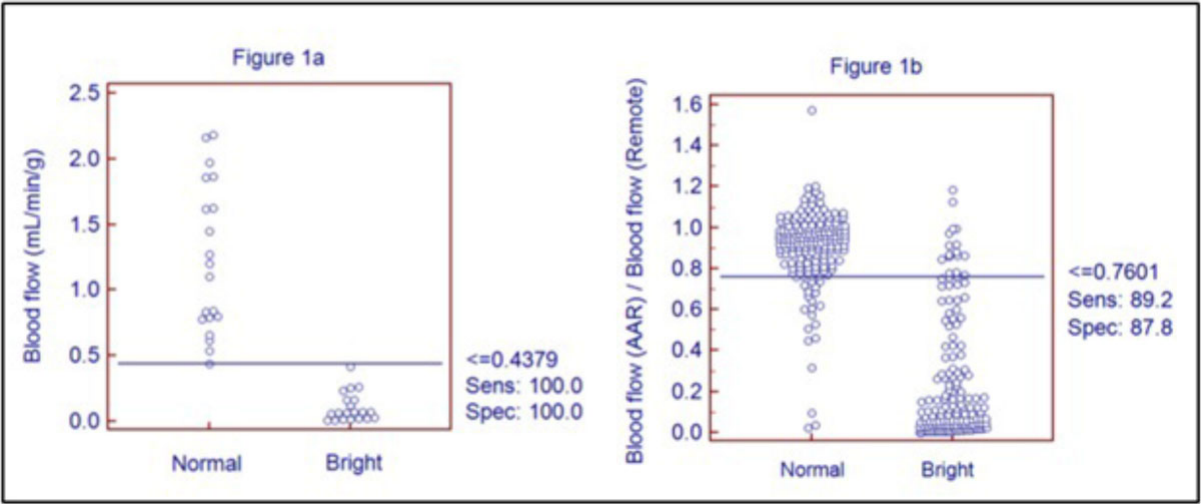
**Absolute myocardial blood flow threshold based on ROI analysis (panel a) and relative myocardial blood flow threshold based on sector analysis (panel b) needed to detect elevated T2 signal intensity**.

In ROI analysis (n = 20), SNR in the AAR was 14.6 +/- 5.7 and CNR of AAR vs. remote regions was 5.7 +/- 2.3. Median blood flow in remote myocardium was 1.15 ml/min/g (IQ 0.73-1.74) and in the AAR 0.072 ml/min/g (IQ 0.020-0.22), p < 0.0001. The blood flow threshold that best separated increased T2 signal intensity from normal was 0.44 ml/min/g (Figure 1a). In a sector-based analysis (n = 320), interobserver agreement for visually detecting T2 signal enhancement was excellent (kappa 0.85, 95% CI 0.79-0.91). The optimal threshold based on the ROC curve was a 24% flow reduction relative to remote (Figure 1b) and an absolute flow < 0.50 ml/min/g. The AUC for relative and absolute blood flow reductions were 0.94 +/- 0.02 and 0.88 +/- 0.02, respectively. Sensitivities and specificities of the three thresholds for detecting T2 enhancement are listed in table 1.

**Table 1:** Sensitivity and Specificity for Determination of T2 Enhancement

Image Analysis	Optimal Threshold	Sensitivity (%)	Specificity (%)
Manual ROI	0.44 mL/min/g	100	100
16 sector matching (absolute flow)	0.50 mL/min/g	78	88
16 sector matching (relative flow )	24% flow reduction	89	88

## Conclusions

A relative reduction in blood flow 24% lower than remote myocardium was the best threshold discriminating detectable T2 enhancement in the area at risk. An absolute blood flow reduction below about 0.44-0.50 ml/min/g also predicted T2 enhancement.

## Funding

Funded by the Intramural Research Program of the National Heart, Lung, and Blood Institute of The National Institutes of Health.

